# Tunable Physical Properties of Electro-Blown Spinning Dextran/Zein Nanofibers Cross-Linked by Maillard Reaction

**DOI:** 10.3390/foods13132040

**Published:** 2024-06-27

**Authors:** Yupeng Ren, Jianhui An, Cheng Tian, Longchen Shang, Yexing Tao, Lingli Deng

**Affiliations:** 1 College of Biological and Food Engineering, Hubei Minzu University, Enshi 445000, China; 202230344@hbmzu.edu.cn (Y.R.); 2020049@hbmzu.edu.cn (J.A.); 1996057@hbmzu.edu.cn (C.T.); 2 Hubei Key Laboratory of Selenium Resource Research and Biological Application, Hubei Minzu University, Enshi 445000, China; 2021021@hbmzu.edu.cn; 3 Hubei Key Laboratory of Biological Resources Protection and Utilization, Hubei Minzu University, Enshi 445000, China

**Keywords:** dextran, zein, electro-blown spinning, Maillard reaction

## Abstract

Electrospinning biopolymer nanofibers have emerged as promising candidates for food packaging applications. In this study, dextran/zein nanofibers were fabricated using electro-blown spinning and subsequently cross-linked via the Maillard reaction (MR) at 60 °C and 50% relative humidity. Compared to traditional electrospinning, the introduction of air-blowing improved the sample preparation speed by 10 times. SEM analysis revealed that the nanofiber morphology remained stable upon MR treatment for 24 h. FTIR spectroscopy confirmed that the MR led to a deformation in the protein conformation and an increase in hydrophilicity and elasticity in the nanofibers cross-linked for 6 h. MR treatment for 18 h considerably enhanced the hydrophobicity and elastic modulus owing to covalent bond formation. Thermal analysis indicated an improved thermal stability with increasing MR duration. Mechanical property analysis revealed an increase in elastic modulus and a decrease in elongation at break for the nanofibers cross-linked for more than 6 h, indicating a trade-off between rigidity and flexibility. Notably, the water vapor permeability of the nanofibers cross-linked for 6 and 18 h was remarkably higher, which can be ascribed to the fiber morphology retention upon water evaporation. Overall, MR-cross-linked dextran/zein/xylose nanofibers showed tunable properties, making them a suitable encapsulation system for bioactive compounds.

## 1. Introduction

In the current context of sustainability and environmental concern, the field of food packaging materials is at a turning point. The development of sustainable and ecofriendly food packaging materials has become crucial as the world grapples with the environmental repercussions of the durability and non-degradability of conventional packaging materials coupled with the alarming generation rates of plastic waste and alarming rates of plastic pollution. The search for alternatives has drawn the attention of researchers toward naturally derived and biodegradable polymers, especially in the context of food packaging. Among these, nanofibers produced using the electrospinning technique have made a notable impact. Electrospinning facilitates the production of extremely fine fibers with improved encapsulation efficiency owing to their high surface-area-to-volume ratios [1].

Zein, a proteinaceous biopolymer isolated from maize with advantageous physicochemical characteristics, has been increasingly recognized for its potential in sustainable food packaging solutions. Zein-based nanomaterials can be prepared using electrospinning technology [2], and various strategies have been explored to enhance the electrospinnability of zein and improve the fiber morphology, mechanical properties, and functionality. For instance, hybrid electrospinning with other biopolymers has enabled the preparation of gelatin/zein [3], cellulose acetate/zein [4], dextran/zein [5], pullulan/zein [6], and gluten/zein [7] nanofibers. Most of the studies on the fabrication of zein-based nanofibers apply traditional electrospinning methods, which result in low yields and are not suitable for industrial applications. Dextran is a hydrophilic, biodegradable biopolymer commonly found in bacterial polysaccharides [8]. Pure dextran nanofibers have been fabricated in DMSO, DMF, or water solutions [9]. Various synthetic polymers has been introduced to reinforce the dextran nanofiber’s properties, such as polylactide-co-glycolide [10,11], polyurethane [12], and polyvinylpyrrolidone [13]. However, few studies have explored the hybrid electrospinning of dextran with biopolymers. In our previous study, the dextran/zein nanofibers were fabricated with a feeding rate of 1.0 mL/h through traditional electrospinning [5]. By addressing the limitation of the yield of traditional electrospinning, our study focuses on improving the fabrication process of nanofibers using electro-blown spinning [14], which is a combination of electrospinning and solution blowing that facilitates nanofiber formation and increases the yield of nanofibers [15,16]. It is applied in air filtration [17,18], yarns [19], batteries [20], nanofiber skin [21], and wound healing [22].

To enhance the performance of biopolymer nanofibers, biopolymer molecules can be modified or combined with additives like nanomaterials, cross-linkers, bioactive compounds, and other polymers, particularly via the Maillard reaction (MR), which is a promising approach to enhance the performance of degradable food packaging materials [23]. The MR is a nonenzymatic browning reaction known for modifying the properties of food materials, such as solubility, wettability, and antioxidant activity. This reaction occurs between amino acids and reducing sugars. In previous studies, high temperatures were applied for the fabrication of MR-cross-linked nanofibers [24,25,26,27,28,29]. Siimon et al. [27] thermally cross-linked glucose-containing electrospun gelatin nanofiber at 170–175 °C between 5 min and 3 h. Kchaou et al. [28] observed a significant decrease in water solubility and an increase in UV barrier ability in gelatin–glucose films heated for 24 h at different temperatures (90–130 °C). Deng et al. [24] fabricated gelatin/zein/glucose nanofibers and cross-linked them in an oven for 3 h at 140 °C. Nevertheless, elevated temperatures yield conjugates, leading to an irremediable loss of functionality and an unregulated proliferation of undesired MR byproducts. Furthermore, high temperatures may compromise the structural integrity of bioactive elements encapsulated within the nanofibers, constricting the application spectrum of high-temperature-glycated nanofibers in the field of active packaging. Therefore, recent studies have focused on the exploration of mild MR conditions. Using mild conditions, Kutzli et al. [30] fabricated a maltodextrin/pea protein isolate and cross-linked it at a temperature of 60 °C and a relative humidity (RH) of 75%, finding the lowest relative amount of free amino acid groups after heating for 24 h. Zhang et al. [7] cross-linked gluten/zein/xylose nanofibers at 60 °C and 40% RH for 24 h, and the cross-linked nanofibers showed improved thermal and water stability. In our previous study [31], gelatin/zein/glucose nanofibers were cross-linked at 60 °C and 50% RH, resulting in improved water resistance and a stiffer network.

In this work, we envisaged that MR under mild conditions might affect the properties of dextran/zein nanofibers to facilitate their application in the encapsulation of bioactive compounds. The objective of this investigation was the fabrication of dextran/zein nanofibers via electro-blown spinning followed by a cross-linking process using the MR under mild conditions. The nanofibers were characterized via scanning electron microscopy (SEM) observation, Fourier transform infrared (FTIR) spectroscopy, and thermal analysis. Water contact angle and X-ray photoelectron spectroscopy (XPS) measurements were performed to analyze the water hydrophilicity/hydrophobicity. The mechanical properties and water vapor permeability (WVP) of the dextran–zein nanofibers were determined to evaluate their potential application in food packaging.

## 2. Materials and Methods

### 2.1. Chemicals

Dextran (MW∼70 kDa) was purchased from Macklin Biochemical Technology Co., Ltd. (Shanghai, China). Zein (grade Z3625, 22–24 kDa) was purchased from Sigma Aldrich (St. Louis, MO, USA). Glucose, xylose, and acetic acid were purchased from Aladdin Reagent Database Inc., Shanghai, China.

### 2.2. Solution Preparation

Dextran (50% *w*/*v*) was dissolved in 50% acetic acid aqueous solution with 0% (*w*/*v*), 5% (*w*/*v*), 10% (*w*/*v*), and 15% (*w*/*v*) zein, respectively. Then, glucose (5% *w*/*v*) or xylose (5% *w*/*v*) was mixed with the above dextran/zein solution. All solutions were stirred overnight to achieve complete dissolution.

### 2.3. Electro-Blown Spinning

**Figure 1:**
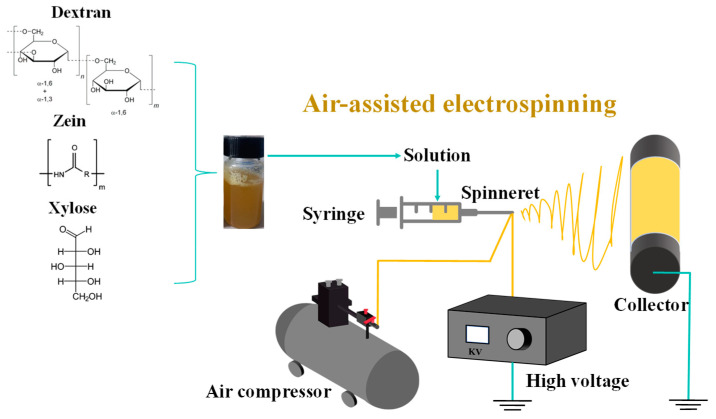
Schematic of the electro-blown spinning setup.

The electro-blown spinning setup used in this study is shown in Figure 1. A 10 mL syringe was used as the solution container, and the solution was continuously pumped to a 20 G needle tip at a speed of 10 mL/h using a pressure pump. The solution was stretched into fibers under an airflow with a flow rate of 400 L/h and an electrostatic field adjusted to 18 kV. The fibers were then collected at 20 cm from the tip.

According to the amount of zein added, the prepared samples were labeled as D50, D50Z5, D50Z10, and D50Z15, respectively. Moreover, the samples added with glucose were labeled as G-D50, G-D50Z5, G-D50Z10, and G-D50Z15, respectively, and those with xylose were labeled as X-D50, X-D50Z5, X-D50Z10, and X-D50Z15, respectively.

### 2.4. Maillard Reaction

The dextran/zein/xylose nanofibrous films were cross-linked via MR, which was conducted in a temperature humidity chamber (HSX-150L, Shanghai Gipp Electronic Technology CO., Ltd., Shanghai, China) at a temperature of 60 °C and an RH of 50% for 0, 3, 6, 12, 18, and 24 h, and the corresponding samples were denoted as M0, M3, M6, M12, M18, and M24, respectively.

### 2.5. Nanofiber Morphology

After vacuum gold-spraying treatment, the morphology of the nanofibers was observed via SEM. Using the Nano Measure 1.2 software, nanofibers were randomly selected from the SEM image, and their diameter distribution was measured.

### 2.6. Colorimetric Measurement

The surface color of the nanofibrous films was assessed using a CS-820N colorimeter (Hangzhou CHNSpec Technology Co., Ltd., Hangzhou, China). The L* (light/dark), a* (red/green), and b* (yellow/blue) color parameters were recorded. The browning index, which represents the purity of the brown color, was calculated according to Gao et al. [32].

### 2.7. FTIR Spectroscopy

The FTIR spectra were recorded using an FTIR spectrometer (iS5, Thermo Nicolet Ltd., Waltham, MA, USA) with the ATR mode over the wavenumber range of 600–4000 cm^−1^ with a resolution of 2 cm^−1^ and 32 scans. According to the method of Siimon et al. [27], the relative absorbance changes (∆RA) of the peaks detected in the spectrum were calculated.

### 2.8. Thermal Properties Analysis

The thermal properties of the nanofibers were analyzed via thermogravimetry analysis (TGA)–differential scanning calorimetry (DSC) (NETZSCH-Gerätebau GmbH, Selb, Germany) using accurately weighed 6–10 mg samples put into a crucible. The empty crucible under the same conditions was used as a reference. The sample was heated from 30 to 600 °C at 10 °C/min under dry N2.

### 2.9. Surface Elemental Analysis

The surface elements of the nanofibers were studied using X-ray photoelectron spectroscopy (XPS) (AXIS SUPRA, Kratos Analytical Inc., Manchester, UK). The spectrum survey scans were obtained over the 0–1350 eV binding energy range at a detector pass energy of 100 eV, and high-resolution spectra were recorded for the C1s region at a pass energy of 50 eV. The survey spectra and the high-resolution spectra of the C1s region were processed using Advantage software, version 5.9931 (Thermo Scientific, East Grinstead, UK) [33].

### 2.10. Water Contact Angle Tests

A tensiometer (OCA 20, Dataphysics Instruments, Filderstadt, Germany) was used to determine the water contact angle of the nanofibers. A precise syringe was used to deposit 3 μL of droplets on the surface of the nanofibers (20 × 40 × 0.2 mm). The contact angle was measured after the droplets contacted the surface of the nanofibers.

### 2.11. Mechanical Properties

The thickness, width, and intercept of the dextran/zein nanofibrous films were tested using a digital micrometer. Then, the tensile strength (TS), elastic modulus (EM), and elongation at break (EB) were measured using a DR-508A (Dongri Instrument Ltd., Dongguan, China) computer tensile testing machine with a load of 5 N. Each sample was measured five times. The following are the calculations for the tensile strength, elongation at break, and elastic modulus [34]:

$$\mathrm{Tensile}\;\mathrm{strength}\;\left(\mathrm{MPa}\right)=\frac{\mathrm{Load}\;\mathrm{at}\;\mathrm{Break}}{\mathrm{Original}\;\mathrm{width}\;\times \;\mathrm{Original}\;\mathrm{thickness}}$$

$$\mathrm{Elongation}\;\mathrm{at}\;\mathrm{break}\;\left(\%\right)=\frac{\mathrm{Elongation}\;\mathrm{at}\;\mathrm{rupture}}{\mathrm{Original}\;\mathrm{test}\;\mathrm{length}}\times 100$$

$$\mathrm{Elastic}\;\mathrm{modulus}\;\left(\mathrm{MPa}\right)=\frac{\mathrm{Stress}}{\mathrm{Strain}}$$

### 2.12. Water Vapor Permeability

The WVP values were assessed utilizing the ASTM E96 gravimetric technique according to the method proposed by Zhang et al. [7], with modifications. The rim of a permeable cup with a capacity of 10 mL was secured with a consistently thick, 6 cm diameter nanofiber film, which was then placed in a desiccator. For all samples, the weights were logged on an hourly basis. This procedure was conducted thrice over 6 h. The WVP was calculated as follows [35]:

$$\mathrm{WVP}\left(g/m\cdot s\cdot \mathrm{pa}\right)=\frac{W_{s}}{\mathrm{At}}\times \frac{L}{∆P}$$

where A represents the contact area (cm^2^), L is the thickness of the nanofibrous film (cm), ∆P refers to the rated vapor pressure differential (Pa) with a set value of 2237.8 Pa at 28 °C, and Ws/t indicates the linear regression of weight over time (g/s).

## 3. Results and Discussion

### 3.1. Fiber Morphologies

**Figure 2:**
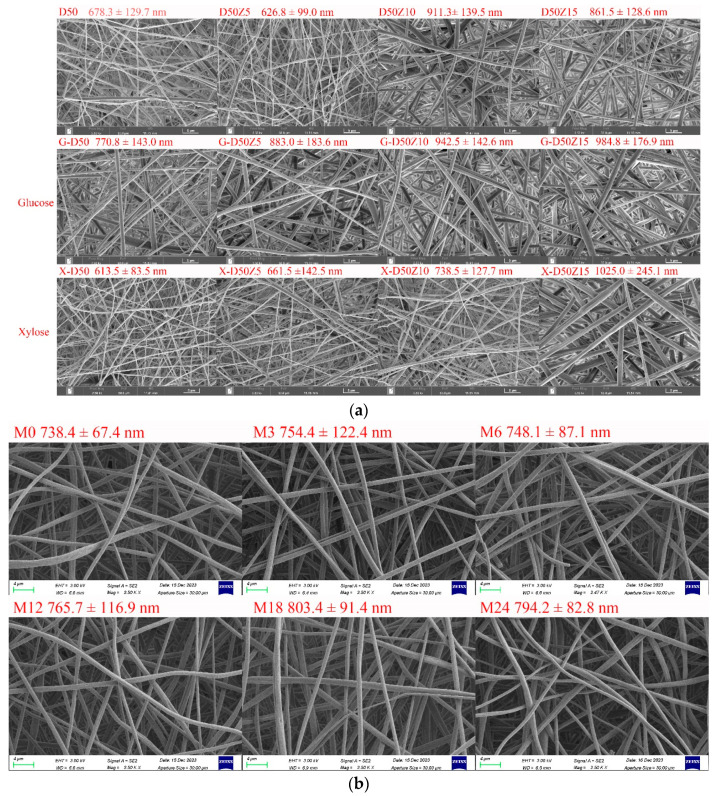
(**a**) SEM images and fiber diameter distributions of dextran/zein nanofibers and those containing glucose or xylose, respectively. D50Z0, D50Z5, D50Z10, and D50Z15 are the nanofibers fabricated using solutions with 50% (*w*/*v*) dextran and 0–15% (*w*/*v*) zein, respectively. G and X refer to the solutions with 5% (*w*/*v*) glucose or xylose. (**b**) X-D50Z5 nanofibers prepared at a temperature of 60 °C and a relative humidity of 50% for 0, 3, 6, 12, 18, and 24 h; the corresponding samples were denoted as M0, M3, M6, M12, M18, and M24, respectively.

Figure 2a shows SEM images of dextran/zein nanofibers with various zein contents and those with glucose or xylose, revealing a nanofiber diameter range of 600–1000 nm (The fiber diameter distributions were supplied in Supplementary Materials Figure S1). In our previous research on the fabrication of dextran/zein via traditional electrospinning, the nanofiber diameter was in the range of 200–700 nm, but the morphology was not very uniform [5]. Conversely, the electro-blown spinning technique provides a more uniform nanofiber morphology and increased electrospinnability. Even though Li et al. [15] found that electro-blown spinning yields thinner nanofibers, other parameters have to be considered. An increase in the flow rate usually increases the fiber diameter [36], which explains the increased diameter of the dextran/zein nanofibers compared with traditional electrospun nanofibers. Moreover, fast-flowing air generates a negative pressure close to the opening of the inner spinning nozzle, which has beneficial effects [37]. Together with the tangential forces, this negative pressure increases the stability of the Taylor cone, inhibits the clogging of needles, and prevents the formation of droplets or beads as the spinning solution exits the nozzle [38]. Compared with traditional electrospinning, air-blowing increases the sample preparation speed by 10 times. In addition, electro-blown spinning is more suitable for the continuous functionalization of large structural components [21].

Figure 2a shows that the nanofiber diameter increased with the increasing zein ratio, and the electrospinnability decreased at a zein content of 15%. Hence, the upper limit of zein solution was set at 15% (*w*/*v*). With the addition of glucose, the dextran/zein nanofiber diameter increased considerably, whereas the addition of xylose did not substantially affect the dextran/zein nanofiber diameter. Considering the electrospinnability, nanofiber diameter, and the fact that xylose is more prone to MR, D50Z5 nanofibers with xylose were selected for further study.

MR can form typical Maillard pigments and result in color variation [39]. Figure 2b shows SEM images and fiber diameter distributions of dextran/zein/xylose nanofibers (X-D50Z5) after cross-linking at 60 °C and 50% RH for 0–24 h. The heat treatment under this condition did not destroy the morphology or porosity of the nanofibers, which was in accordance with our previous results [31]. The retention of the nanofiber morphology and surface area-to-volume after cross-linking facilitates the application of the nanofibers as an effective encapsulation system for bioactive compounds. Table 1 presents the color change of the dextran/zein nanofibers after the MR. The a* (redness/greenness) and b* (yellowness/blueness) values increased with the heating time. The color changed nearly linearly with the heating time from 0 to 12 h, causing the formation of low-molecular-weight pigments. For the nanofibers heated for 18 h, the yellowness decreased slightly. Sun et al. [40] found that increasing the temperature considerably increases the browning intensity, which is accompanied by reduced lightness (L*), a substantial loss in yellowness (+b*), and a shift toward redness (+a*).

### 3.2. FTIR Spectroscopy Analysis

**Figure 3:**
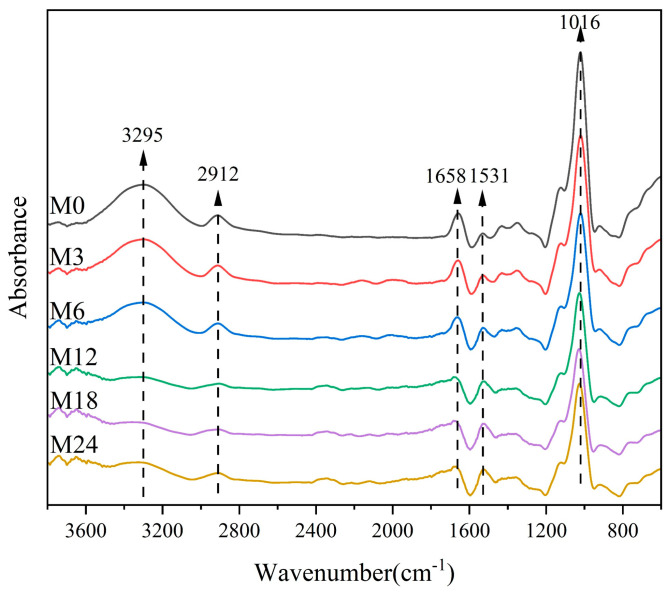
FTIR spectra of nanofibers before 0 (M0) and after cross-linking for 3 h (M3), 6 h (M6), 12 h (M12), 18 h (M18), and 24 h (M24).

Figure 3 shows the FTIR spectra of the dextran/zein/xylose nanofibers before and after heating for 3–24 h. The band at 3295 cm^−1^ corresponds to the stretching vibration of free hydroxyl groups and amino groups. Table 1 shows that the relative absorbance of this band decreased with the heating time, which can be ascribed to the decreased ratio of hydroxyl groups and free amino groups during the MR. The band at approximately 2924 cm^−1^ is due to the –CH_2_ stretching vibration of aliphatic groups [41]. The bands centered at 1658 and 1531 cm^−1^ can be assigned as amide I and amide II bands, respectively. The relative absorbance of the band at 1658 cm^−1^ may also represent the vibration from the Shiff base because there is an overlap of C=O and C=N vibration bands [42]. The band corresponding to C–O stretching is centered at 1016 cm^−1^. Table 2 lists the relative absorbance of the typical peaks in the FTIR spectra. After the MR, the relative absorbance at 1016 and 1038 cm^−1^ decreased with the heating time, which can be attributed to the glycation between the carbonyl group of xylose and the free amino acid group of zein [27]. Siimon et al. [27] observed that the amide I band underwent the largest change in relative absorbance during cross-linking of gelatin/glucose, which was accompanied by a decrease in the peaks at 1081 and 1035 cm^−1^ associated with C–O vibrations, mainly in glucose. A similar change trend was also observed in our previous study [31].

### 3.3. Thermal Analysis

**Figure 4:**
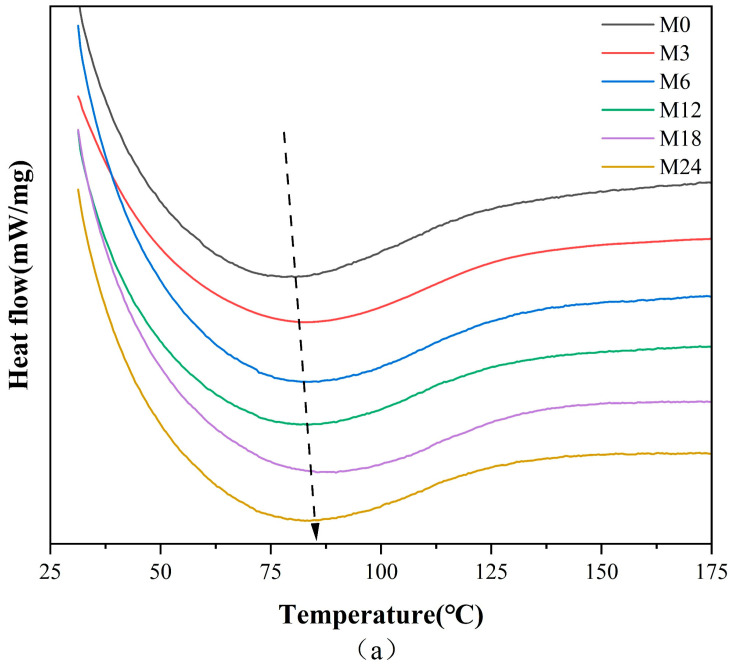

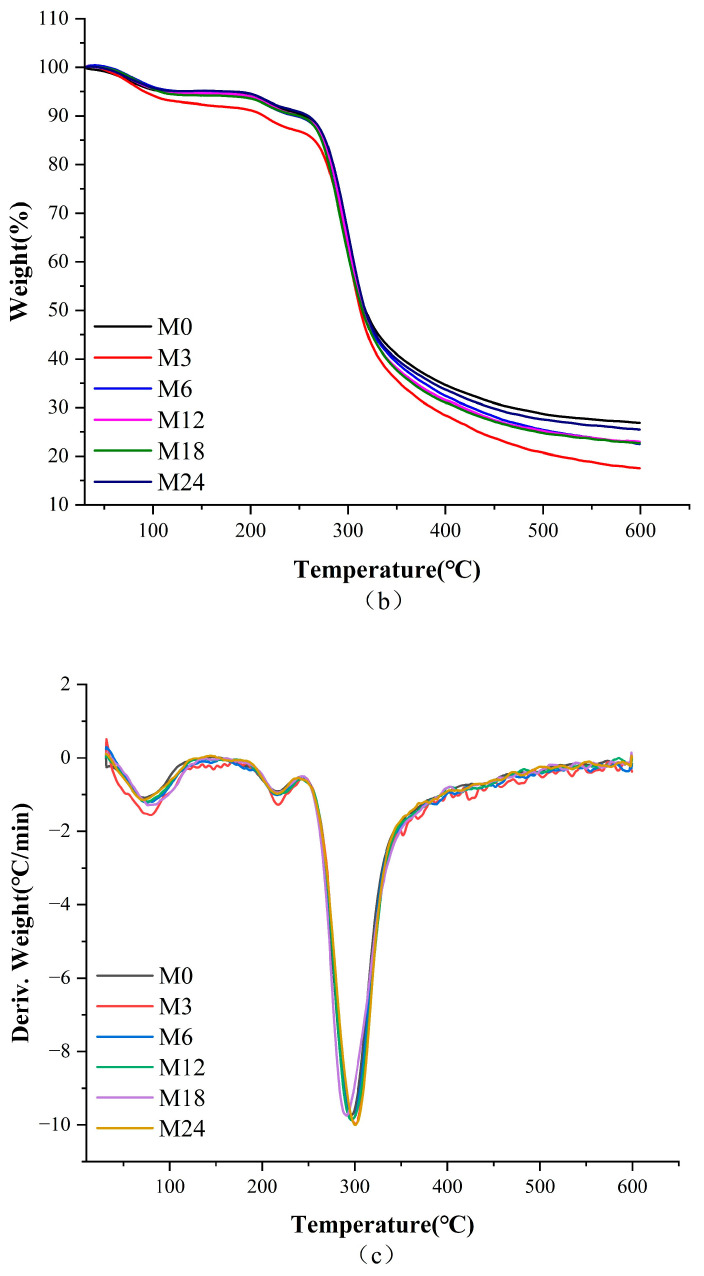
Thermodynamic analysis diagram before 0 (M0) and after cross-linking for 3 h (M3), 6 h (M6), 12 h (M12), 18 h (M18), and 24 h (M24): (**a**) DSC curves; (**b**) TGA curves; (**c**) TGA derivative curves.

Figure 4 illustrates the DSC, TGA, and TGA derivative curves of the nanofibers before and after the MR, and the corresponding thermal data are shown in Table 3. The denaturation temperature displayed a gradient increase from 80.6 °C for M0 to 88.9 °C for the M18 nanofibers and a decrease to 83.8 °C for the M24 nanofibers. This increase in denaturation temperature suggests an enhancement in the thermal stability against denaturation of the nanofibers, indicating the inception of the MR between protein and xylose. The color metrics indicate that during the heating phase of 0–12 h, low molecular pigments were formed within the nanofibers. Conversely, nanofibers subjected to 24 h of heating likely resulted in the generation of high molecular pigments, decreasing the heat stability of the nanofibers.

Table 3 shows that the heat degradation of nanofibers primarily occurred in three stages. The primary phase can be largely ascribed to the expulsion of bound water from the nanofibers. The second peak can be ascribed to the decomposition of the proteins [3]. The third peak corresponded to the decomposition of the nanofibers. The decomposition temperature increased slightly from 296.1 °C for M0 to 298.3 °C and 297.4 °C for M3 and M6, respectively, indicating that the stability of the nanofibers improved with increasing cross-linking time.

### 3.4. Water Contact Angle Analysis

**Figure 5:**
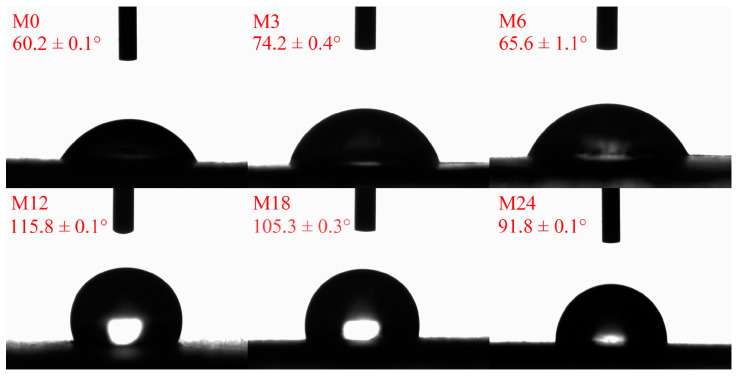
Water contact angles before 0 (M0) and after cross-linking for 3 h (M3), 6 h (M6), 12 h (M12), 18 h (M18), and 24 h (M24).

The hydrophobicity/hydrophilicity of a nanofibrous film is an important factor for the application of nanofibers. Figure 5 illustrates the water contact angles of the dextran/zein/xylose nanofibrous films before and after the MR. Before the MR, the dextran/zein/xylose nanofibers exhibited a hydrophilic surface and were easily dissolved by water. After heating for 3 and 6 h, the water contact angles increased slightly. Further heating for 12 and 18 h increased the water contact angle to 115.8° and 105.3°, respectively. For the nanofibrous film heated for 24 h, the water contact angle decreased slightly compared with that of M18. The decreased water contact angle for M24 was associated with the formation of degraded hydrophilic small molecules, which may increase the hydrophilicity of the nanofibrous film. Previous studies have also reported that MR cross-linking can increase the hydrophobicity of nanofibrous films, improving their water stability [24,31].

### 3.5. Surface Elemental Analysis

**Figure 6:**
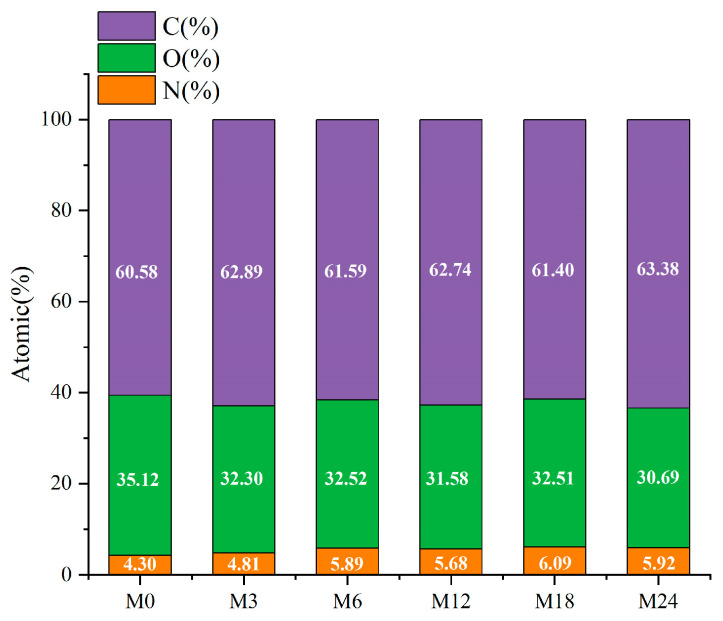
Elemental compositions (atomic%) before 0 (M0) and after cross-linking for 3 h (M3), 6 h (M6), 12 h (M12), 18 h (M18, and 24 h (M24).

**Figure 7:**
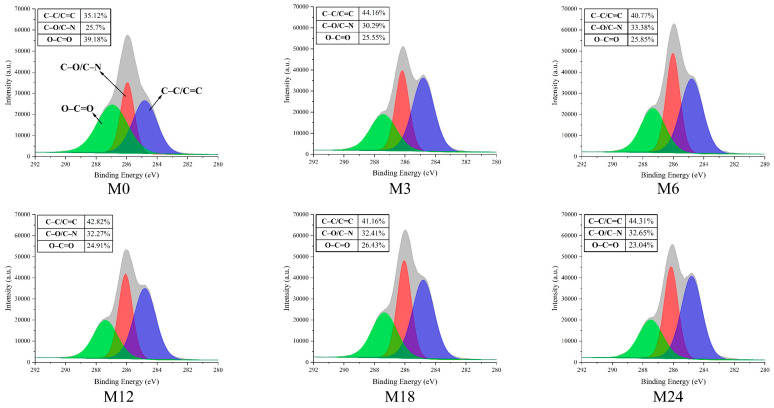
High-resolution C1s XPS spectra before 0 (M0) and after cross-linking for 3 h (M3), 6 h (M6), 12 h (M12), 18 h (M18), and 24 h (M24).

The surface property of the nanofibrous films was further analyzed through XPS, and the results are presented in Figure 6 and Figure 7. Figure 6 shows that the elemental composition of the nanofibrous films changed after the MR. The proportion of C increased slightly and that of O decreased with increasing heating time. The increased ratio of N indicates an increase in the ratio of protein exposed on the nanofiber surface. The hydrophobic nature of zein may increase the water contact angles and retard the wetting process of the nanofibrous film.

The high-resolution C1s spectra displayed in Figure 7 reveal three distinct peaks at 284.7, 286.2, and 288.0 eV, which correspond to C–C/C=C, C–O/C–N, and O–C=O, respectively [43]. The proportion of C–C/C=C increased from 35.12% (M0) to 44.16% (M3) and then to 40.77% (M6), 42.82% (M12), 41.16% (M18), and, finally, 44.31% (M24). In accordance with the increase in hydrophobicity of the nanofibrous films after the MR, the proportion of O–C=O decreased with prolonged heating time.

### 3.6. Mechanical Property Analysis

**Figure 8:**
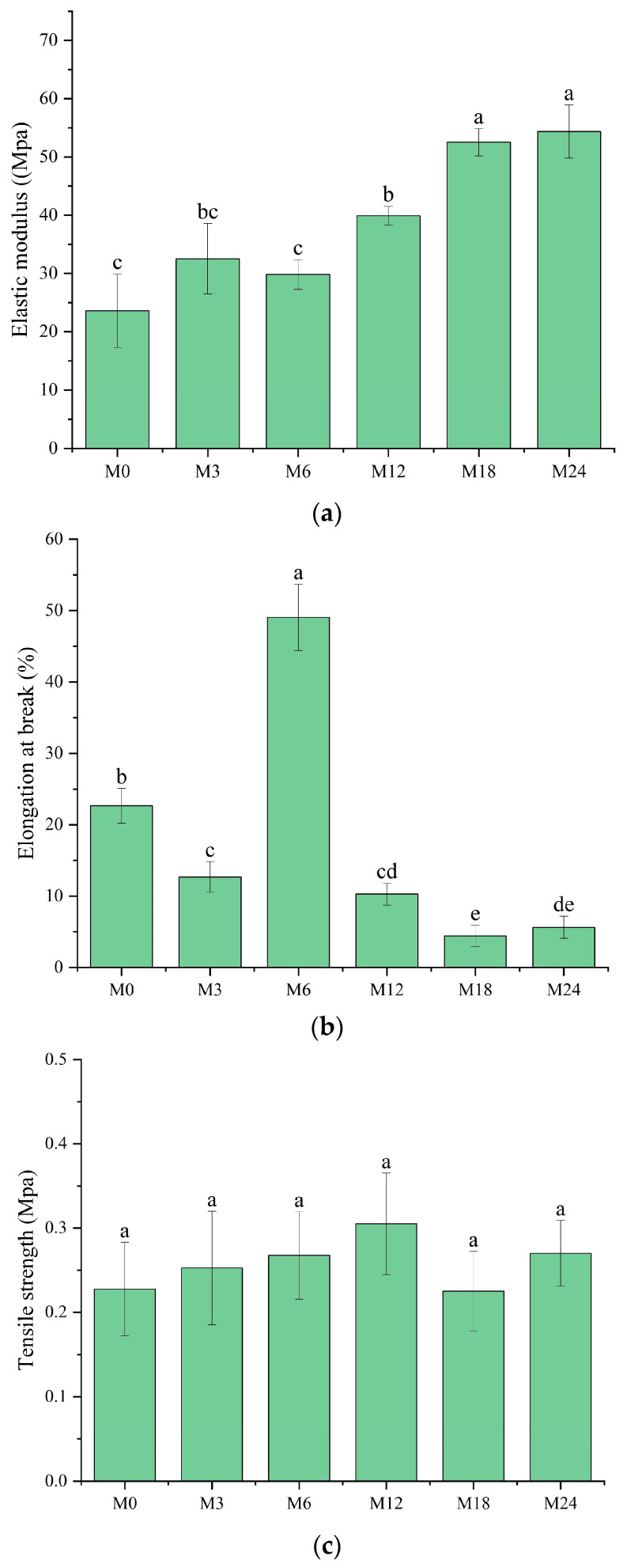
Mechanical properties before 0 (M0) and after cross-linking for 3 h (M3), 6 h (M6), 12 h (M12), 18 h (M18), and 24 h (M24): (**a**) elastic modulus; (**b**) elongation at break; (**c**) tensile strength. Different letters indicate significant difference (*p* < 0.05) between samples.

A tensile test was conducted to analyze the mechanical properties of the dextran/zein/xylose nanofibrous films before and after MR. The elastic modulus (Figure 8a), elongation at break (Figure 8b), and tensile strength (Figure 8c) of the nanofibrous films were determined. The elastic modulus remained the same after cross-linking for 3 and 6 h. Meanwhile, cross-linking for 12, 18, and 24 h substantially increased the elastic modulus to 39.89, 52.51, and 54.36 MPa, respectively, which can be attributed to the dense structure formed upon protein cross-linking with xylose, i.e., the intermolecular entanglements and interactions between polymer chains [44]. However, the formation of cross-linked structures decreased the polymer chain mobility, as indicated by the decreased elongation at break for M12, M18, and M24. Interestingly, the nanofibrous film cross-linked for 6 h exhibited a markedly higher elongation at break (49.05%), indicating the higher flexibility of the molecules within the nanofibers. We assume that at this stage the conformation of the proteins started to unfold, and xylose may also act as a plasticizer in the nanofibers at the first stage of the cross-linking [45].

### 3.7. WVP Analysis

**Figure 9:**
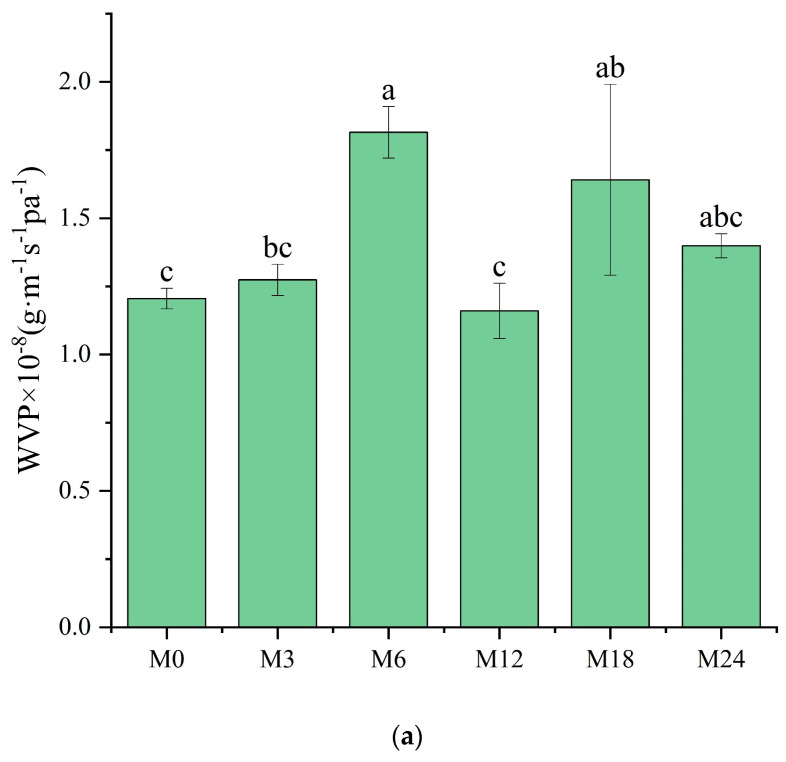

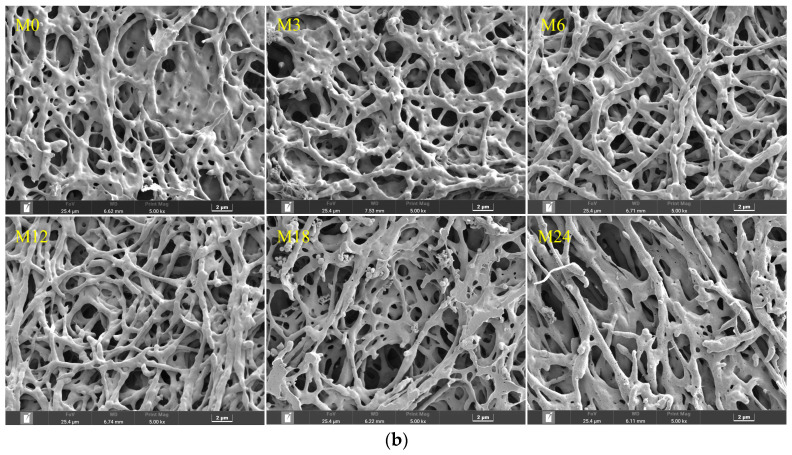
(**a**) Water vapor permeability before 0 (M0) and after cross-linking for 3 h (M3), 6 h (M6), 12 h (M12), 18 h (M18), and 24 h (M24); (**b**) SEM images of the nanofibers after immersion in water for 24 h. Different letters indicate significant difference (*p* < 0.05) between samples.

The WVP of films is one of their most important properties to determine their applicability as packaging materials. Fresh products need to be packaged in films with high WVP, whereas processed foods require films with low WVP for long-term storage. As shown in Figure 9a, compared with M0, M6 and M18 exhibited a substantially higher WVP. However, no marked changes were observed for the other samples. WVP depends on the absorption and diffusion mechanisms of water vapor. These mechanisms are intimately connected to the composition, specifically the ratio of hydrophilic to hydrophobic components, and the architecture of polar and nonpolar groups [35,46]. The inherent hydrophilicity of substances, such as dextran and xylose, promotes water-induced swelling in nanofibers, thereby decreasing the porosity, which may retard the diffusion of water vapor, as shown by the decreased WVP. However, the MR between the protein and xylose increased the hydrophobicity, as indicated by the water contact angle results. The increased hydrophobicity of the nanofibrous film facilitated the retention of the nanofiber morphology and porosity when water vapor diffused through the film, which can explain the increased WVP of the cross-linked nanofibrous film. Liu et al. [31] also observed a remarkably increased WVP of a gelatin/zein/glucose nanofibrous film after MR cross-linking. Figure 9b exhibits SEM images of the nanofibers after immersion in water for 24 h. The M0 and M3 nanofibers agglomerated, and the porous structure was lost after water treatment. After the MR cross-linking, the porous structures of M6, M12, and M18 were maintained. In wound dressing application, a high WVP is conducive to the wound exudate. Hence, the cross-linked dextran/zein nanofibers might find application as a delivery system for fresh food packaging or wound healing dress.

## 4. Conclusions

In this work, uniform dextran/zein/xylose nanofibers were fabricated via electro-blown spinning, which considerably improved the yield of nanofibers compared with traditional electrospinning. The nanofibers were cross-linked for 0–24 h, resulting in tunable physical properties. The nanofibers cross-linked for 6 h (M6) showed increased elongation at break, and those cross-linked for more than 6 h exhibited substantially decreased elasticity, hindering their manipulation during application. The notable increase in WVP in M6 compared with M0 is due to the enhanced stability of the nanofibers upon cross-linking, which prevents dissolution during vapor penetration. Under the conditions of vapor passage, the ability of M6 to maintain a relatively high porosity is favorable for future applications in the encapsulation of bioactive substances because they could operate in an area with a high surface area ratio. The improved yield compared to traditional electrospinning methods considerably optimizes manufacturing efficiency. The ability to manipulate the physical properties of nanofibers by cross-linking for varying durations opens up possibilities for tailored product development based on specific requirements, increasing the versatility of uses across different industry sectors. The cross-linked dextran/zein nanofibers are well-suited for encapsulation of bioactive substances, aiding usage in systems where controlled release or targeted delivery is pivotal. A larger surface area ratio also works to the advantage of leveraging the bioactivity of these substances, leading to desirable responses in bioactive systems.

## Figures and Tables

**Table 1:** Color parameters and browning index (BI) of xylose/dextran/zein nanofibers after cross-linking for 0, 3 h, 6 h, 12 h, 18 h, and 24 h.

Sample	L* (Lightness)	a (Redness/Greenness)	b (Yellowness/Blueness)	BI (Browning Index)
M0	97.050 ± 0.040 ^b^	−0.370 ± 0.010 ^d^	2.273 ± 0.015 ^f^	2.559 ± 0.011 ^f^
M3	97.013 ± 0.035 ^b^	−0.380 ± 0.01 ^cd^	2.353 ± 0.006 ^e^	2.649 ± 0.005 ^e^
M6	97.327 ± 0.031 ^a^	−0.393 ± 0.006 ^c^	2.547 ± 0.006 ^d^	2.846 ± 0.008 ^d^
M12	97.103 ± 0.065 ^b^	−0.417 ± 0.006 ^b^	2.693 ± 0.012 ^c^	3.020 ±0.012 ^b^
M18	97.373 ± 0.015 ^a^	−0.420 ± 0.01 ^b^	2.663 ± 0.006 ^b^	2.983 ± 0.005 ^c^
M24	97.300 ± 0.026 ^a^	−0.443 ± 0.006 ^a^	2.970 ± 0.001 ^a^	3.316 ± 0.005 ^a^

Values in the same column followed by different superscripts are significantly different (*p* < 0.05).

**Table 2:** Relative absorbance of FTIR spectra before 0 (M0) and after cross-linking for 3 h (M3), 6 h (M6), 12 h (M12), 18 h (M18), and 24 h (M24).

Wavenumber/cm^−1^	3271	2887	1653	1540	1459	1348	1271	1110	1038	1016
M0	4.01%	2.20%	5.41%	1.60%	2.40%	3.81%	3.61%	11.82%	25.45%	39.68%
M3	8.00%	4.38%	6.10%	3.81%	4.76%	5.71%	5.14%	11.24%	23.43%	27.43%
M6	7.75%	3.97%	6.24%	3.78%	4.73%	5.67%	4.91%	11.34%	24.01%	27.22%
M12	8.22%	4.11%	6.92%	4.30%	5.23%	6.17%	5.42%	11.40%	22.24%	25.98%
M18	8.40%	3.80%	7.80%	4.80%	6.00%	6.40%	5.60%	11.20%	21.00%	25.00%
M24	8.35%	3.97%	8.35%	5.43%	6.26%	6.26%	5.64%	10.86%	20.46%	24.43%

**Table 3:** DSC and TGA data before 0 (M0) and after cross-linking for 3 h (M3), 6 h (M6), 12 h (M12), 18 h (M18), and 24 h (M24).

	DSC		TGA						
	T (°C)	∆H (J/g)	Peak 1 (°C)	Weight Loss (%)	Peak 2 (°C)	Weight Loss (%)	Peak 3 (°C)	Weight Loss (%)	Residue at 600 °C (%)
M0	80.6	−29.086	73.3	5.14	216.0	4.61	296.1	63.39	26.86
M3	82.8	−27.752	76.5	7.81	214.7	5.93	298.3	68.88	17.38
M6	83.1	−31.134	76.3	5.43	216.1	4.40	297.4	67.86	22.47
M12	83.8	−30.141	76.4	5.26	215.9	4.19	296.4	67.58	22.96
M18	88.9	−28.588	76.5	5.82	217.7	3.87	291.1	67.62	22.69
M24	83.8	−30.192	71.1	4.84	216.2	3.91	300.2	65.78	25.47

## Data Availability

The original contributions presented in the study are included in the article and Supplementary Material, further inquiries can be directed to the corresponding authors.

## Supplementary Materials

The following supporting information can be downloaded at: https://www.mdpi.com/article/10.3390/foods13132040/s1, Figure S1: (a) Fiber diameter distributions of the dextran/zein nanofiber and that with glucose or xylose. D50Z0, D50Z5, D50Z10, D50Z15 indicated the nanofibers fabricated from solutions with 50% (*w*/*v*) dextran and 0–15% (*w*/*v*) zein respectively. The G or X indicated the solutions with 5% (*w*/*v*) glucose or xylose. (b) X-D50Z5 nanofibers prepared at temperature of 60 °C and relative humidity of 50% for 0, 3, 6, 12, 18, and 24 h, and the corresponding samples were denoted as M0, M3, M6, M12, M18, and M24, respectively.
