# Supplementary material for: Tunable Physical Properties of Electro-Blown Spinning Dextran/Zein Nanofibers Cross-Linked by Maillard Reaction
Source: Foods. 2024 Jun 27;13(13):2040. doi: 10.3390/foods13132040 (PMC11241757; doi:10.3390/foods13132040)
Supplement: Supplementary file 1 [file foods-13-02040-s001.zip › foods-3056973-supplementary.pdf]

Supplementary Materials

Tunable physical properties of electro-blown spinning dextran/zein nanofibers cross-linked by Maillard reaction

Yupeng Ren [1], Jianhui An [1], Cheng Tian [1], Longchen Shang [2], Yexing Tao [1,*] and Lingli Deng [2,3,*]

1 College of Biological and Food Engineering, Hubei Minzu University, Enshi 445000, Hubei;

2 Hubei Key Laboratory of Selenium Resource Research and Biological Application（Hubei Minzu University）, Enshi 445000, Hubei

3 Hubei Key Laboratory of Biological Resources Protection and Utilization（Hubei Minzu University）, Enshi 445000, Hubei

* Correspondence: 2023060@hbmzu.edu.cn (Y.T.); 2019040@hbmzu.edu.cn (L.D.)

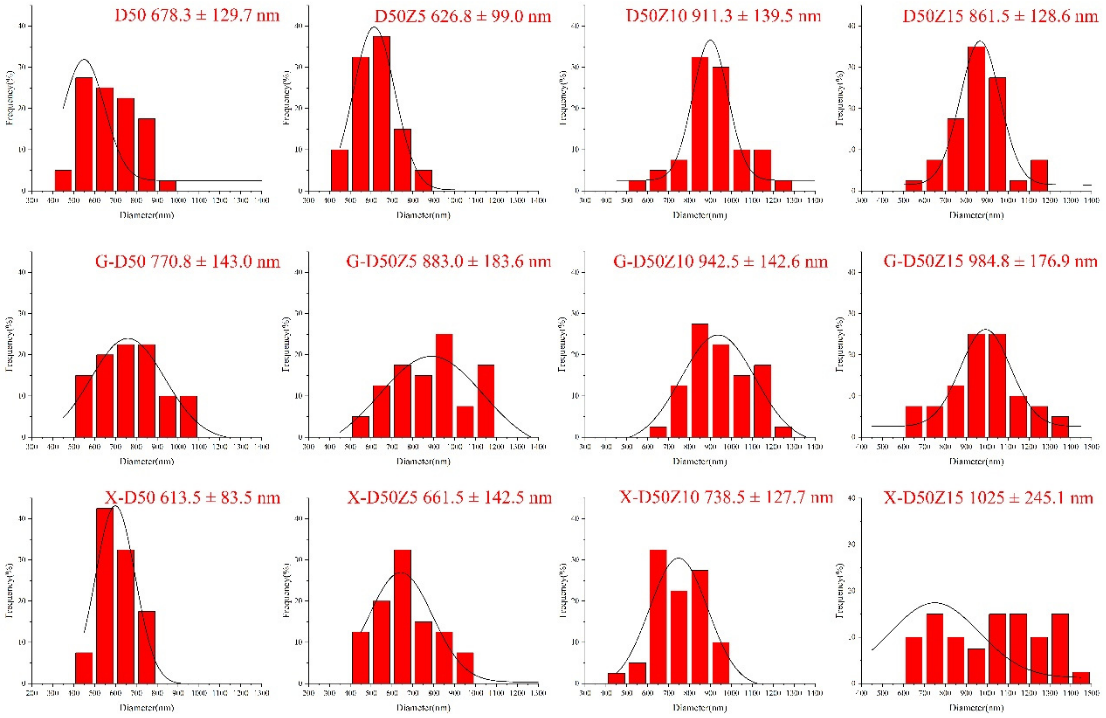

(a)

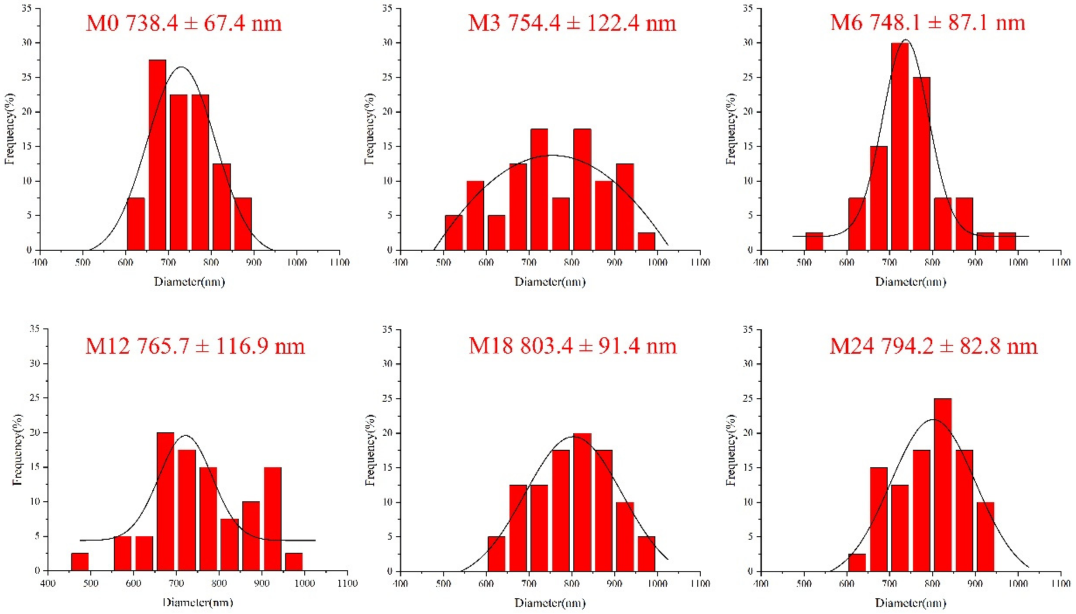

(b)

Figure S1. (a) Fiber diameter distributions of the dextran/zein nanofiber and that with glucose or xylose. D50Z0, D50Z5, D50Z10, D50Z15 indicated the nanofibers fabricated from solutions with 50% (w/v) dextran and 0-15% (w/v) zein respectively. The G or X indicated the solutions with 5% (w/v) glucose or xylose. **(b)** X-D50Z5 nanofibers prepared at temperature of 60 °C and relative humidity of 50% for 0, 3, 6, 12, 18, and 24 h, and the corresponding samples were denoted as M0, M3, M6, M12, M18, and M24, respectively.
